# Simplified inelastic electron tunneling spectroscopy based on low-noise derivatives

**DOI:** 10.1038/s41598-022-21302-4

**Published:** 2022-11-10

**Authors:** Shankar Kesarwani, Shobhna Misra, Dipankar Saha, Maria Luisa Della Rocca, Indrajit Roy, Swaroop Ganguly, Ashutosh Mahajan

**Affiliations:** 1 Department of Electrical Engineering, Indian Institute of Technology Bombay, Mumbai, India; 2 Université Paris Cité, CNRS, Laboratoire Matériaux et Phénomènes Quantiques, 75013 Paris, France; 3 Spaceage Geoconsulting, Banks, ACT 2906 Australia; 4 Centre for Nanotechnology Research, Vellore Institute of Technology, Vellore, 632014 India

**Keywords:** Characterization and analytical techniques, Characterization and analytical techniques, Nanosensors, Electrical and electronic engineering

## Abstract

A standard experimental setup for Inelastic Electron Tunneling Spectroscopy (IETS) performs the measurement of the second derivative of the current with respect to the voltage ($d^2I/dV^2$) using a small AC signal and a lock-in based second harmonic detection. This avoids noise arising from direct differentiation of the current-voltage characteristics (I–V) by standard numerical methods. Here we demonstrate a noise-filtering algorithm based on Tikhonov Regularization to obtain IET spectra (i.e. $d^2I/dV^2$ vs. V) from measured DC I–V curves. This leads to a simple and effective numerical method for IETS extraction. We apply the algorithm to I–V data from a molecular junction and a metal-insulator-semiconductor tunneling device, demonstrating that the computed first/second derivatives have a workable match with those obtained from our lock-in measurements; the computed IET spectral peaks also correlate well with reported experimental ones. Finally, we present a scheme for automated tuning of the algorithm parameters well-suited for the use of this numerical protocol in real applications.

## Introduction

Inelastic Electron Tunneling Spectroscopy (IETS) is a powerful tool to measure vibrational spectra, introduced by Lambe and Jaklevic in 1966^1^. Since then, it has been widely used for studying electron-phonon inelastic interaction and vibrational spectroscopy of molecules and organic and inorganic thin films^2–6^. Other well-established vibrational spectroscopy techniques, like Raman Spectroscopy and Fourier Transform Infrared Spectroscopy (FTIR), require somewhat involved optical setups. IETS, on the other hand, is an all-electronic technique, and as such does not exclude the optically inactive modes of vibration. It also promises ultra-high sensitivity and high selectivity^7–10^.

In 1996, a new dimension to IETS was opened up with the proposition from Luca Turin that biological olfaction might be based on sensing the vibrational energies of odorant molecules through an IETS mechanism^11^. While controversial, this ‘Vibration Theory of Olfaction’ gave a new lease of life to the longstanding hypothesis that smell is derived from molecular vibrations^12^. It also positioned Olfaction as a prototypical system in the nascent field of Quantum Biology^13^.

This proposition has piqued technological interest in electronic nose (e-nose) sensors, which would be biomimetic in the sense of being IETS-based^14,15^. Currently, there is an abundance of e-nose technologies for gas or vapour sensing, based on various transduction mechanisms (e.g. resistive, capacitive)^16,17^; but mimicking natural olfaction is still an open challenge. A biomimetic sensor would be a significant advance with potential applications in multiple domains, e.g. security (as can be seen from the use of sniffer dogs to detect explosives and drugs), environmental monitoring, healthcare, food/agriculture, and cosmetics.

**Figure 1:**
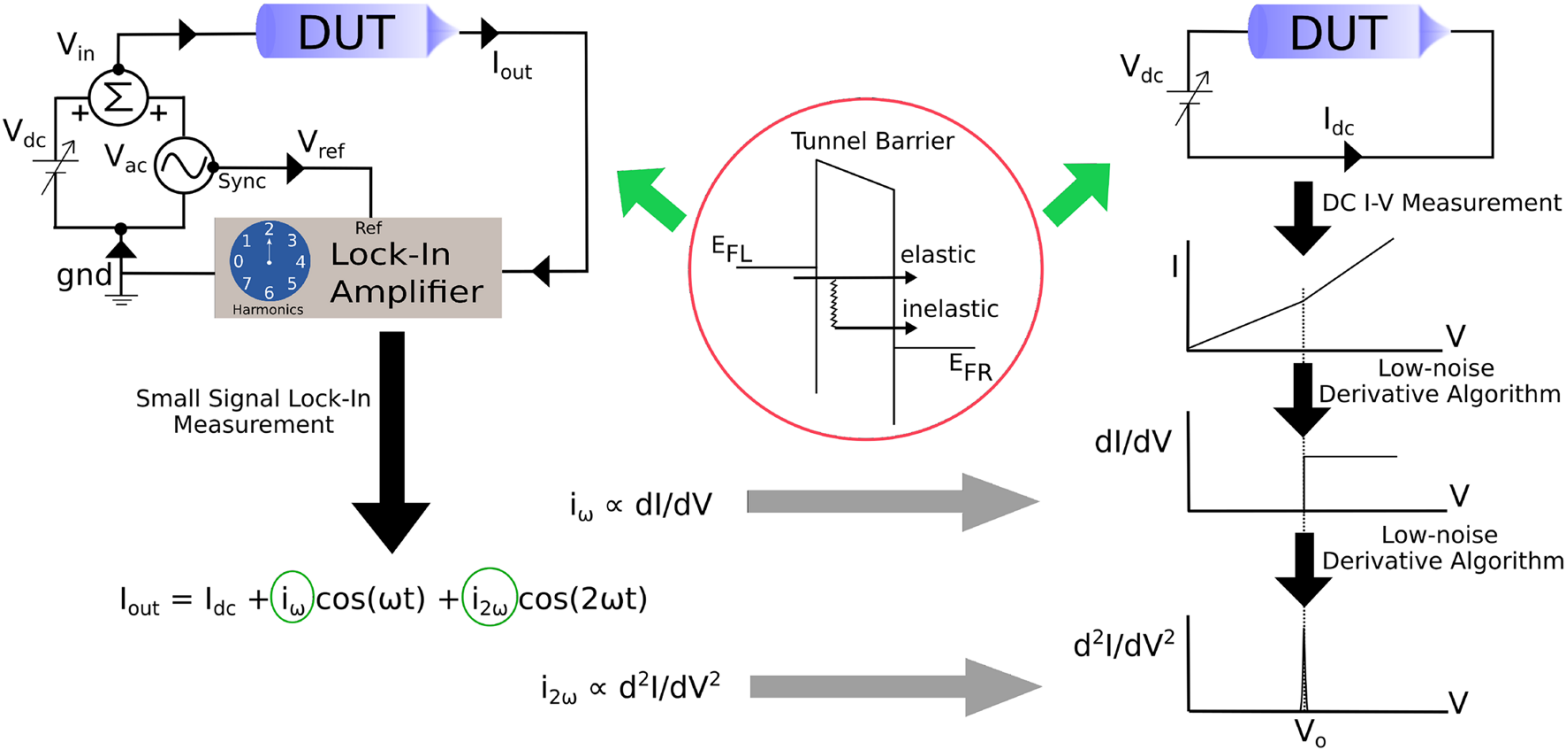
Schematic of IETS phenomenology (center), the standard setup thereof (left), and the method proposed here (right). The inset in the central red circle shows a tunnel barrier connected to contacts. The opening up of the inelastic channel leads to a kink in the I–V characteristics, as seen in the top panel of the stacked schematic plot on the right. The first derivative gives a step-like feature, and the second derivative a spectroscopic peak as shown underneath. Since usual numerical differentiation is noisy, the setup used for IETS is a small-signal lock-in illustrated on the top left. It applies a combined dc plus ac input to the tunneling ‘device under test’ (DUT). The output current I$_{\hbox {out}}$ will contain dc and ac components, including higher harmonics. The first (second) harmonic will be proportional to the first (second) derivative, and these are detected by a lock-in amplifier. Our setup, illustrated on the top right, features a simple I–V measurement followed by a filtering algorithm to generate low-noise derivatives. This leads to the IET spectrum in the intuitive way illustrated by the stacked plots on the right.

Thus, IETS could be a lab tool for physico-chemical analysis and, with further development, a plausible candidate to realize a quantum biomimetic e-nose sensor.

The process of inelastic electron tunneling occurs when electrons tunneling through a thin barrier lose part of their energy in exciting phonon modes in the barrier or vibrational modes of proximate molecules. The onset of such inelastic tunneling process is indicated by a kink in the current vs. voltage (I–V) characteristics whenever q times V (where q is the electronic charge) increases beyond the energy of a vibrational mode. This corresponds to the opening of an inelastic tunneling transport channel as shown in the red circle of Fig. 1. The vibrational spectra depend on the constituent atoms and their bondings, as a consequence it acts as a chemical fingerprint of the barrier material. Since the elastic current usually dominates over the inelastic contribution in most tunneling devices, signatures of inelastic transport are hard to discern directly in the I–V curves. This can be better addressed by measuring or computing the second derivative of the I–V curves. This leads to a vibrational spectrum (the IET spectrum) with peaks corresponding to various vibrational modes, similar to standard Raman or FTIR spectra. However, traditional methods of computing numerical derivatives based on finite differences, typically lead to large noise levels. This difficulty is surmounted in a typical IETS experimental setup for a direct second derivative measurement. In this case, under an applied small AC input voltage, the non linear tunneling device current output is composed by higher order harmonics of the applied input; Taylor Series analysis shows the second harmonic to be proportional to the second derivative of the I–V curve. Homodyne detection is used to measure the second harmonic of the output signal. However, lock-in amplifiers, while versatile and robust for the derivative measurement, make the experimental setup bulky and expensive, particularly for e-nose sensor applications.

In this work, we have implemented a filtering algorithm to obtain low-noise first and second derivative computation from noisy DC I–V measurements. This alternative protocol can be highly suited for the realization of compact IETS-based sensor systems, as well as analytical setups. It can also greatly simplify low-noise derivative measurements for a larger range of other applications^18,19^. We validate our approach by comparing our results with standard IETS measurements performed on two devices of different nature, a molecular junction and a metal-insulator-semiconductor (MIS) tunneling device.

The novelty here springs from the adaptation of the filtering algorithm (hereafter called ‘Algorithm’) from the applied mathematics literature^20,21^. We introduce the “Algorithm” section. Its application to reproduce the experimental results from traditional IETS measurement on a molecular junction^22^ is presented in “Results and discussions: molecular junction” section. In “Results and discussions: MIS device” section, we similarly show its application to IETS for a MIS tunneling device. In this case, the IETS peaks are seen to be merged with each other; we show that vibrational peak decomposition enables the identification of the material system nonetheless. In “Automated computation of IET spectra” section, we present a strategy to automate the tuning of the parameters of the Algorithm for the estimation of the low-noise derivatives of I–V, which would be a necessary element of a real analytical setup or sensor system.

## Algorithm

IET spectra consist of peaks in the second derivative of I vs. V at voltages (or equivalently energies or wavenumbers) corresponding to the excited vibrational modes. We present a far simplified approach to IETS numerical computation based on a direct evaluation of a low-noise first and second derivative of the I–V curve. The experimental and numerical approaches are schematically compared in Fig. 1. On the left, the typical experimental set-up is illustrated, where DC and AC voltage signals are added up and fed to the device under test (DUT); the device current $I_{out}$ goes as the input to a lock-in amplifier, for detection of its first and second harmonic components. The right part of Fig. 1 shows the schematic of the DC I–V measurement setup. The proposed noise filtering algorithm needs only the measured current as function of the bias voltage in order to calculate the first and second derivative of the I–V curve, analogous to the experimental approach. The small-signal measurement with a lock-in tuned at the first harmonic yields the first derivative of I vs. V, i.e. the conductance; thus the filtering Algorithm yields a first derivative en route to the second. We have therefore, in all cases, also compared these two as an intermediate validation of the Algorithm.

Let vector *y* be the measured experimental data for the current and *x* be its derivative, the conductivity. They are related by the matrix equation $\varvec{A}x=y$, where $\varvec{A}$ is the assembled matrix by finite difference scheme with a quadrature rule of integration.

The low-noise derivative estimation here is essentially a noise filtering process based on Tikhonov Regularization^20^. It imposes the following costs—mathematically parameterized in a ‘Cost Functional’, $J_{\eta }(x)$, as seen in Eq. (1). One, for moving away from the simple derivative of the given noisy function—this is the ‘Fidelity’ term $\phi$ in the Cost Functional. Two, for a derivative that is too jittery—the ‘Penalty’ term $\psi$ in the Cost Functional,

1 $$\begin{aligned} J_{\eta }(x)= \phi (\varvec{A}x-y) +\eta \psi (x), \end{aligned}$$

which can be written in matrix form as,

2 $$\begin{aligned} J_{\eta }(x)= (\varvec{A}x-y)^T(\varvec{A}x-y)+ \eta \varvec{D} x^T \varvec{D} x, \end{aligned}$$

where, $\eta$ is the tuning parameter, widely known as a regularization parameter that is essentially the outcome of the Tikhonov regularization^20^.

The regularization parameter $\eta$ is determined by the balancing principle as described in^21^. The balance between these terms is attained by the Regularization Parameter $\eta$ and a second parameter $\gamma$ that controls the shape of the solution. The Penalty and Fidelity terms can be related linearly as follows^21,23^:

3 $$\begin{aligned} \psi (x)=\gamma \eta \phi (x) \end{aligned}$$

The mathematical details of the Algorithm, including the balancing principles for the determination of the Regularization Parameter, are detailed in Supplementary Material, Section I. The flow chart therein, Fig. S1, illustrates the steps in the method for obtaining the low-noise second derivative of the I–V characteristics. In the following sections, this method has been applied to calculate IET spectra for two different systems, which match very good those obtained from a standard IETS setup. Lastly, we mention in passing that this filtering Algorithm has also been implemented and validated on a Raspberry Pi electronic microcontroller.

## Results and discussions: molecular junction

The Algorithm has been tested on IETS results reported by Salhani *et.al*^22^ on a large-area vertical molecular junction. These junctions are based on a cross-conjugated Anthraquinone (AQ) layer. The junctions were fabricated in a standard cross-bar geometry and the molecular layer was covalently grafted on the base electrode by diazonium electrochemistry. The total AQ thickness was estimated to $\sim$8 nm by atomic force microscopy (AFM).

Low-noise transport measurements were carried out at low temperature ( 5 K) by acquiring simultaneously the DC current-voltage characteristics, its first and second derivative, by the lock-in technique. The rms modulation voltage chosen for the measurements was 8 mV at a frequency of $f=$ 17 Hz, and the integration time was 3 sec. The longer integration time reduces noise levels. Noise reduction is also effected by utilizing two low-pass filter options in the lock-in amplifier: the input analog filter and the synchronous filter. Signatures of vibrational modes excited by inelastic events are revealed in the whole measured voltage range.

**Figure 2:**
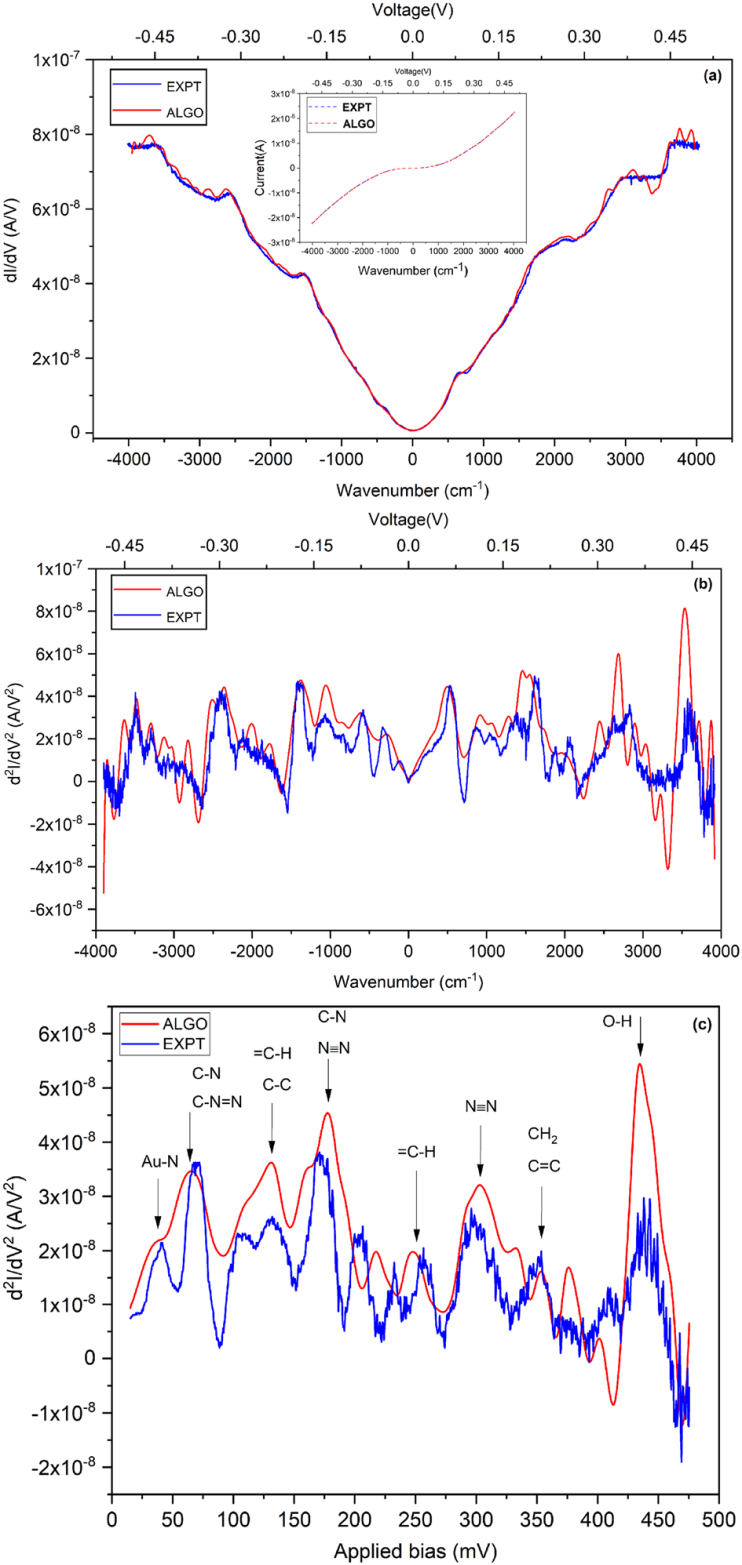
Blue and red colors denote, respectively, plots obtained from experiment (EXPT) and by the application of the filtering algorithm (ALGO) for a molecular junction. (**a**) First derivative of the current as a function of voltage (conductance), dI/dV vs. V. Inset: I–V characteristics. (**b**) Second derivative of the current as a function of voltage (IET spectrum), d$^{2}$I/dV$^{2}$ vs. V. (**c**) Both positive and negative bias IETS peaks are represented as curves plotted after Symmetrization on the experimental data and algorithm curves.

The Algorithm is applied to the I–V characteristics measured experimentally as described in Ref.^22^. The Algorithm generates the first and second derivative as well as the filtered I–V data, as seen in Fig. 2a,b. Both the first and second derivative functions produced by the algorithm are compared with the direct experimental measured data in Fig. 2. A good matching is obtained for the algorithm parameter $\gamma$ equal to 0.8. That includes the multiple peaks in the second derivative (IETS) data as seen in Fig. 2(c) where the peak assignmens to vibrational modes have been indicated.

**Figure 3:**
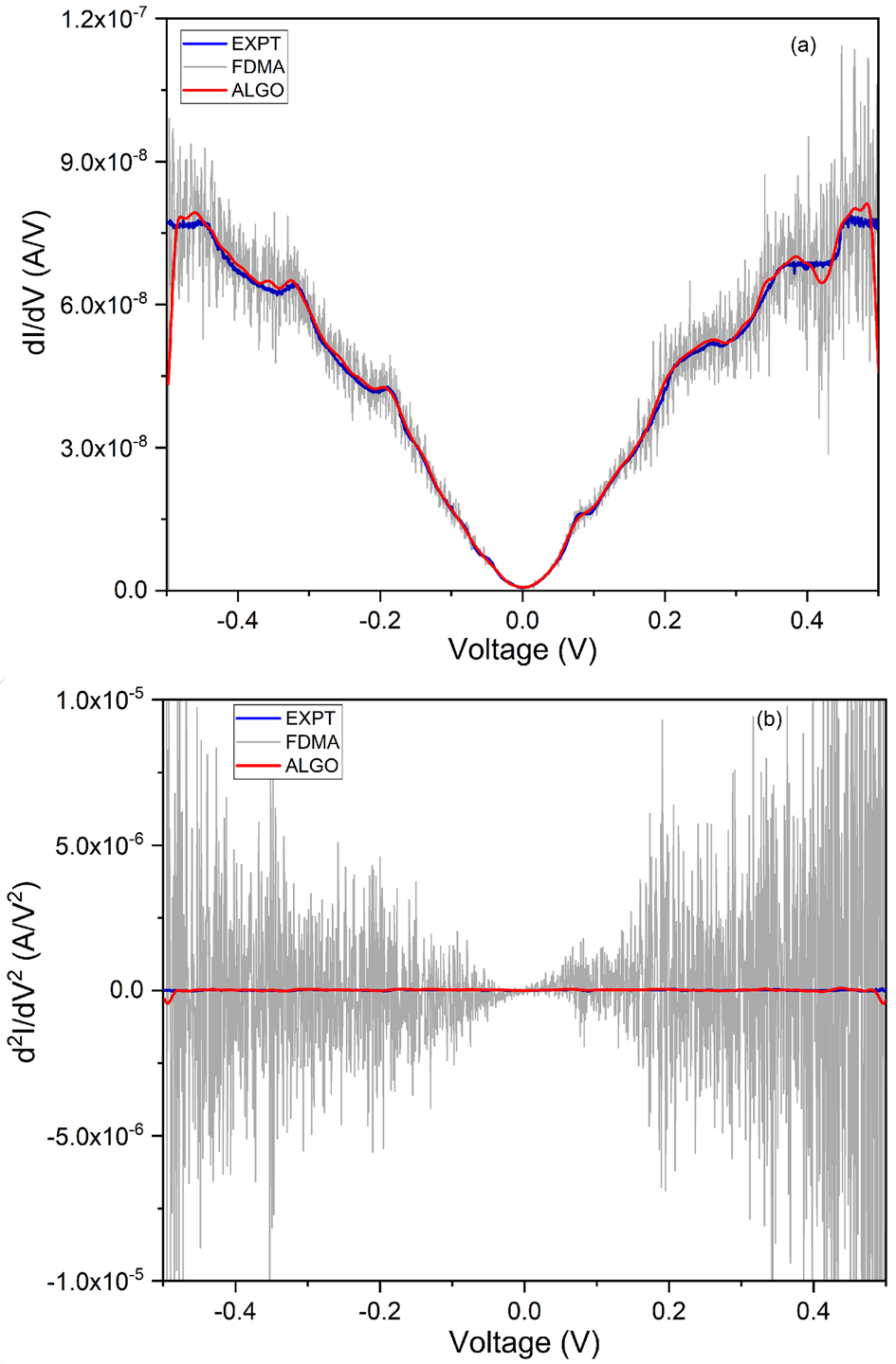
Comparison of derivatives of current from: experimental lock-in measurement (EXPT, in blue); our filtering algorithm (ALGO, in red); and, finite differences smoothed by moving averages (FDMA, in gray). (**a**) First derivative of current with respect to voltage, i.e. conductance, vs. voltage. The FD first derivative has been filtered with a moving average of width 5. (**b**) Second derivative of current with respect to voltage vs. voltage, i.e. IET spectrum. The FD second derivative has been filtered with a moving average of width 9.

In Table 1, we bring out the difference in the peak position obtained by the lock-in measurement and by the algorithm. The error in detection is calculated for the quantitative characterization. It can be seen that the error in peak position is less than 4$\%$ except for the second peak and the peaks detected by the algorithm fall in the range reported in experimental measurements in^22^ and references therein. However, 3 peaks out of 13 reported in the experimental measurements are missed out by the algorithm. The first and second derivatives calculated using the standard finite difference method with moving average smoothing (FDMA) are shown for comparison in Fig. 3. The first derivative is smoothed with a moving average of width 5, but is still significantly noisier, particularly at higher bias. Whereas the second derivative, despite smoothing with a moving average of width 9, is extremely noisy.

Lastly, we note that the DC I–V characteristics here are measured while applying the AC modulation voltage. This could lead to a broadening of spectral peaks as in the lock-in measurement, due to the modulation voltage. In hindsight, the accuracy could be improved by removing the AC modulation during the DC measurement.

This constitutes the first validation of our approach of using the filtering derivative Algorithm on DC I–V data, as a promising alternative to the standard IETS setup.

## Results and discussions: MIS device

**Figure 4:**
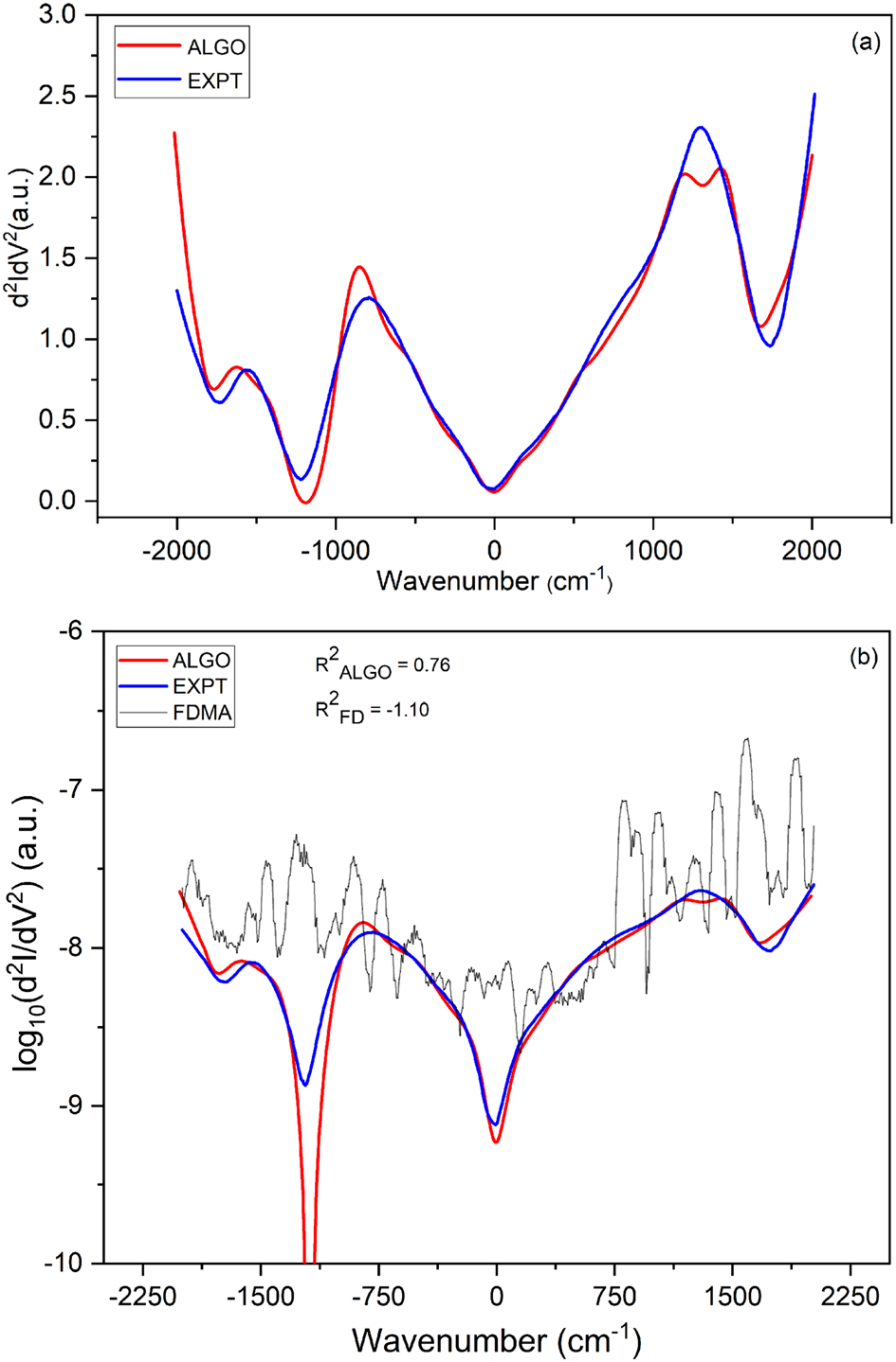
Comparison of second derivative of current with respect to voltage vs. wavenumber, i.e. IET spectrum—from smoothed experimental lock-in based second-harmonic measurement (EXPT, in blue); from our filtering algorithm (ALGO, in red); and from finite differences smoothed by moving averages (FDMA, in gray). (**a**) Linear-linear plot of IET spectrum for MIS tunnel, showing EXPT and ALGO. (**b**) Log-linear plot of IET spectrum, showing EXPT, ALGO, and FDMA. The FD second derivative has been filtered with a moving average of width 9 here. R$^{2}$ is the coefficient of determination, quantifying the match to EXPT.

The second test of the Algorithm has been performed on MIS tunneling devices, fabricated for the purpose of this study. The MIS comprises a 10/100 nm thick Cr/Au as the top metal, followed by 2 nm layer of $\hbox {HfO}_{2}$ from Atomic Layer Deposition (ALD) as the insulating layer and Si(100) substrate with n-type doping of about 10$^{19}$ /cm$^{3}$. The IETS measurements were performed at a temperature of 10 K. The device fabrication and measurement are detailed in Supplementary Material, Section II. The Algorithm is applied to the measured DC I–V to obtain dI/dV vs. V. A comparison of the first derivatives is shown in the Supplementary Material, Section II. Applying the Algorithm to this dI/dV then yields d$^2$I/dV$^2$ vs. V. A comparison of the second derivatives (IETS) is shown in Fig. 4 with the same color code of Fig. 2. In both cases, the derivative spectra produced by the Algorithm matches well with those obtained from the direct measurements. In comparison, we see from Fig. 4b that the second derivative calculated using the standard finite difference method is extremely noisy even after smoothing with a moving average of width 9 (FDMA). The coefficient of determination is computed for both the Algorithm and the FDMA method, in order to quantify their accuracy.

**Figure 5:**
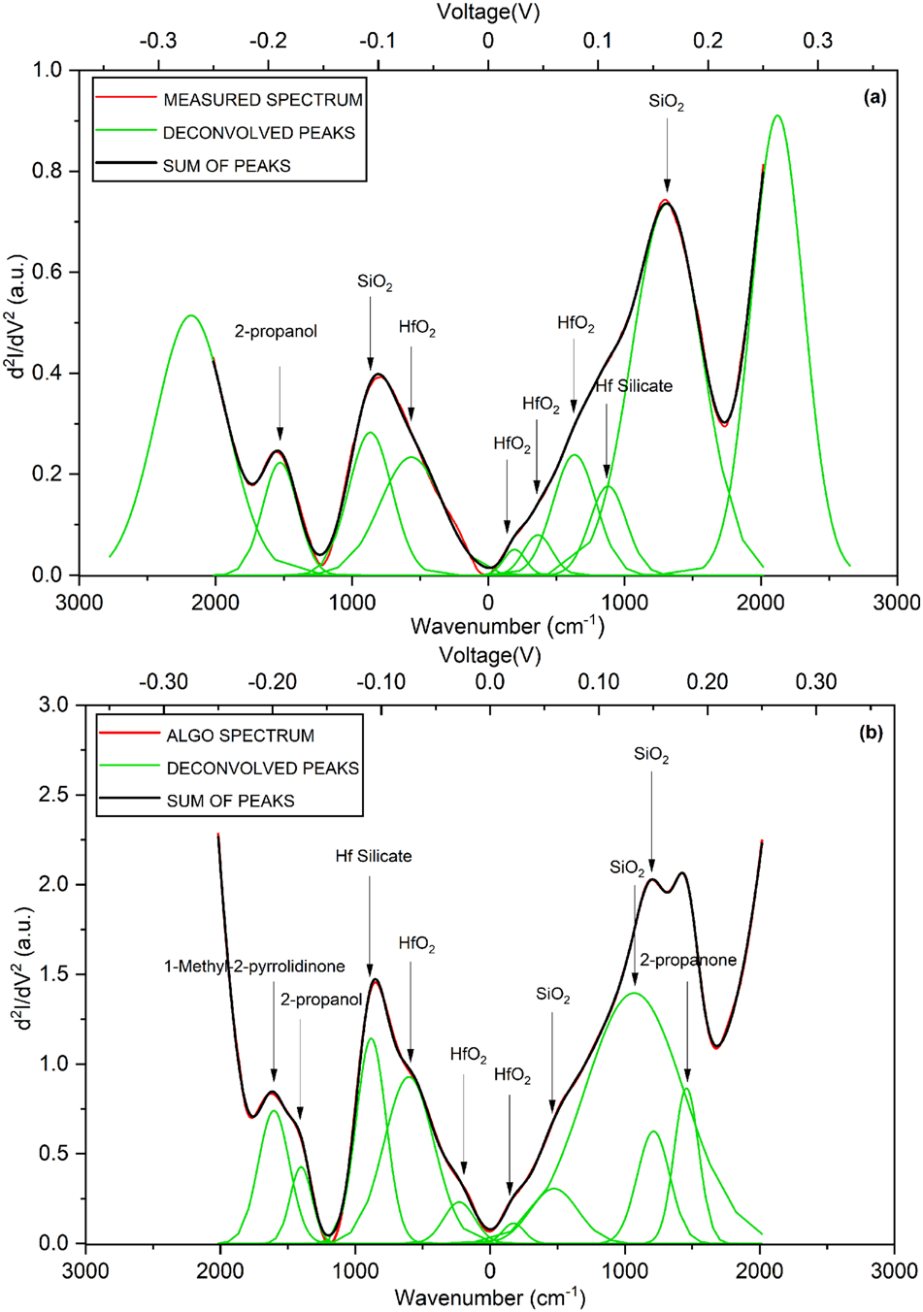
Deconvolution of IET spectra separating out the peaks related to molecular vibration identified by the arrows for MIS tunneling device. Red denotes the IET spectrum, green denotes the deconvoluted peaks, and black denotes their sum (which match with the IET spectra). (**a**) Experimental IETS, from small-signal lock-in measurement. The peaks at 193, 364, 565, 633 cm$^{-1}$ belong to HfO$_2$, peaks at 865, 1310 cm$^{-1}$ belong to SiO$_2$, 879 cm$^{-1}$ to Hf Silicate and 1527 cm$^{-1}$ corresponds to 2-propanol. (**b**) IETS obtained by application of the filtering algorithm. The peaks at 174, 229, 601 cm$^{-1}$ belong to HfO$_2$, peaks at 475, 1069, 1212 cm$^{-1}$ belong to SiO$_2$, 882 cm$^{-1}$ to Hf Silicate, 1402 cm$^{-1}$ corresponds to 2-propanol, 1457 cm$^{-1}$ to 2-propanone and 1603 cm$^{-1}$ to 1-Methyl-2-pyrrolidinone.

The device structure is not symmetrical due to which there is an asymmetry between positive and negative bias spectra^4,24^. The IET spectra present overlapping peaks. The identification of the molecular vibration related to chemical species would require to deconvolve these peaks inferring the contributions which add up to give the spectrum. Obviously, this is an optimization problem that involves finding out the peak characteristics (e.g. number, shape, center, height, width) so that their superposition gives the best fit to the IETS spectrum. Note that peaks overlapping is a known problem affecting IETS measurements at high temperatures. Thus, a robust tool for the deconvolution of overlapping peaks could also enable higher-temperature IETS numerical analysis. We carried out unconstrained deconvolution (i.e. without any user-specified peak values) of the IET spectra obtained from the Algorithm, and from the lock-in measurement and is plotted in Fig. 5. Peak identification in terms of the responsible molecule/material and mode was carried out to the best of available information (previously reported Raman, FTIR and IET spectroscopic data), and is reported in Table 2. Peaks obtained from the experimental IETS at 193, 364, 565, 633 cm$^{-1}$ correspond to the Monoclinic $\hbox {HfO}_{2}$, peaks at 865, 1310 cm$^{-1}$ belong to $\hbox {SiO}_{2}$ molecule (Asymmetric Stretch), 879 cm$^{-1}$ for Hf-silicate and 1527 cm$^{-1}$ for 2-propanol are in good agreement with the literature^4,24–27^. Similarly the peaks obtained from the Algorithm at 174, 229, 601 cm$^{-1}$ belong to Monoclinic $\hbox {HfO}_{2}$, peaks at 475 cm$^{-1}$ to $\hbox {SiO}_{2}$ molecule (Rocking), peaks at 1069, 1212 cm$^{-1}$ to $\hbox {SiO}_{2}$ molecule (Asymmetric Stretch), 882 cm$^{-1}$ for Hf-silicate, 1402 cm$^{-1}$ for 2-propanol, 1457 cm$^{-1}$ for 2-propanone and 1603 cm$^{-1}$ for 1-Methyl-2-pyrrolidinone. We note that the errors between peaks obtained here versus those previously reported is well within 10 percent in almost all cases^4,25–29^. In principle, the error could be further minimized by reducing the peak width through the insertion of RF filters in the measurement lines^30–33^. Now, we point out that $\hbox {HfO}_{2}$, $\hbox {SiO}_{2}$, Hf Silicate and 2-propanol are adequately identified by both the techniques, though the spectra obtained are not exactly identical. This is promising for the development of this and similar algorithms, indicating that with sufficient data and training, they can eventually replace the small-signal second harmonic approach traditionally used in IETS experiments. A preliminary approach toward that is described in the following section.

**Table 1:** IETS peaks obtained from standard measurements and from the filtering algorithm; identified through comparison with peaks reported in^22^ and references therein).

S. no.	Energy in meV (lock-in measurement)	Energy in meV (algorithm)	Error(%) (deviation from the mean)	Assignment
1	6–18	NOT detected	–	AQ skeletal def
2	25–40	37	13.85	$\nu$(Au–N)
3	60–75	65	3.7	$\nu$(C–N) $\delta$(C–N=N)
4	100–110	NOT detected	–	$\delta$(=C–H) $\delta$(N–H)
5	125–135	130	0	$\delta$(=C–H) $\nu$(C–C)
6	165–180	177	2.60	AQ ring stretch.$\nu$(C–N), $\nu$(N=N)
7	200–210	205	0	AQ aromatic def.$\nu$(C=C), $\nu$(C=O)
8	230–235	234	0.64	$\delta$(=C–H) overtones
9	250–265	247	4.0	$\delta$(=C–H) overtones
10	285–310	302	1.51	$\nu$(N$\equiv$N)
11	345–360	353	1.41	$\nu$(C=C) overtones $\nu$(CH2)
12	400–415	NOT Detected	–	$\nu$(N–H), $\nu$(O–H) $\nu$(C=O) overtones $\nu$(C=C)
13	427–450	434	1.02	$\nu$ (O–H)

**Table 2:** IETS peaks obtained from standard measurements and from the filtering algorithm; identified through comparison with peaks reported in the literature from IET, Raman and FTIR spectroscopy.

Molecule/material (mode)	Spectroscopy techniques	Peaks reported (cm^−1^)	IETS ALGO (cm^−1^)	Error (%) ALGO (cm^−1^)	IETS EXPT(cm^−1^)	Error (%) EXPT (cm^−1^)
$\hbox {HfO}_{2}$ Monoclinic	RAMAN	175	174	0.57	193	10.29
$\hbox {HfO}_{2}$ Monoclinic	RAMAN	241	229	4.98	–	–
$\hbox {HfO}_{2}$ Monoclinic	IR, RAMAN	355, 355	–	–	364	2.54, 2.54
$\hbox {HfO}_{2}$ Monoclinic	RAMAN, IETS	577, 584	–	–	565	2.08, 3.25
$\hbox {HfO}_{2}$ Monoclinic	RAMAN, IETS	639,640	601	5.95, 6.09	633	0.94, 1.09
$\hbox {SiO}_{2}$ (Rocking)	IR	457	475	3.94	–	–
$\hbox {SiO}_{2}$ (Asymmetric Stretch)	IETS	847	–	–	865	2.13
$\hbox {SiO}_{2}$ (Asymmetric Stretch)	IR	1076	1069	0.65	–	–
$\hbox {SiO}_{2}$ (Asymmetric Stretch)	IR	1256	1212	3.50	1310	4.30
Hf silicate	IETS	928	882	4.96	879	5.28
$^{*}$2-propanol	IR	1466	1402	4.37	1527	4.16
$^{*}$2-propanone	IR	1420	1457	2.61	–	–
$^{*}$1-Methyl-2-pyrrolidinone	IR	1674	1603	4.24	–	–

Note that the available data allows identification of the molecule/material in all cases, but not always the vibrational mode. (*These molecules are conjectured to be organic residues from our sample processing).

## Automated computation of IET spectra

The filtering Algorithm balances ‘Fidelity’ to the nominal derivative of a noise-added function with a ‘Penalty’ for an excessively jittery derivative, in order to arrive at the optimal estimate of the true derivative. Now, for the Algorithm to be used as a standalone tool for low-noise derivative computation, we need to develop a method for automatic parameter estimation. In particular, we focus on automating the choice of the parameter $\gamma$ in Eq. (3).

First, in order to expand our working data-set of IETS-like spectra beyond the experimental ones from the use cases above, we generate a synthetic dataset, as suggested by Ito et al.^21^. The synthetic I–V data is obtained by twice integrating a sum of Gaussian peaks (corresponding to noise-free IETS) and adding white (Additive White Gaussian) or colored (pink and brown) noise thereto. The detailed methodology has been elucidated in the Supplementary Material, Section III.

We find that the optimal $\gamma$ depends on a few factors. Namely, the magnitude of the additive noise; and, any two of the following: the resolution between data points, their number, the range of the data. Automation of the choice of $\gamma$ is accomplished by fitting analytical formulas to $\gamma$ as a function of the relevant parameters.

**Figure 6:**
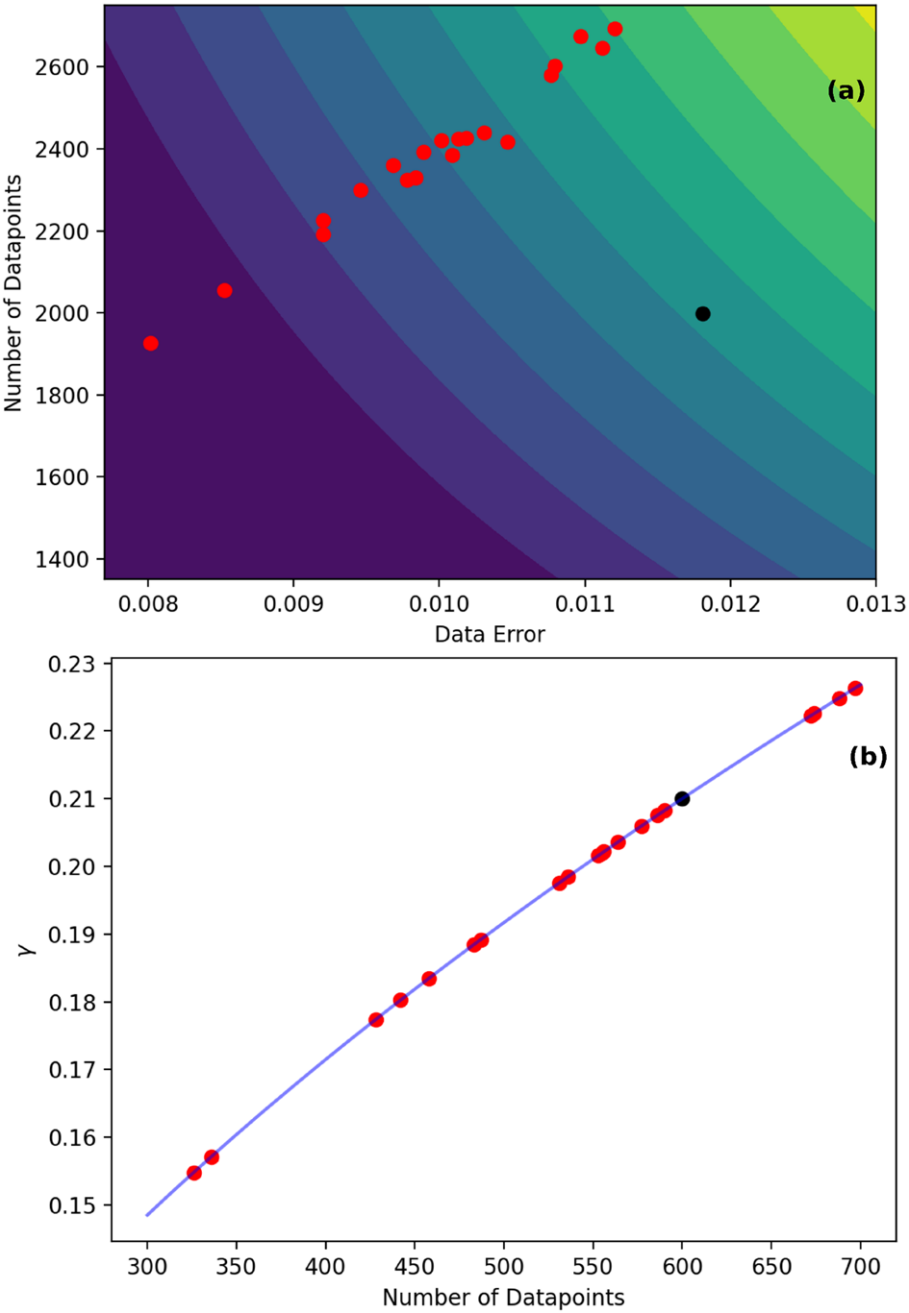
(**a**) Contour plot of surface showing the relation of $\gamma$ with the noise to signal ratio and number of points. Red points show synthetic data and black point corresponds to experimental data. (**b**) Plot showing the relation of $\gamma$ with the number of points, red points show synthetic data and the black point corresponds to experimental data.

The resolution of the I–V data on the voltage scale plays an essential role in the choice of $\gamma$ since a finer resolution captures smaller-scale features. In this case, the formula for $\gamma$ needs more inputs (than for a coarser resolution) for optimal fitting. For voltage resolution below 0.5 mV, we find that the optimal $\gamma$ must be considered to depend on both the noise level and the number of points in (or equivalently, the data range of) the spectrum. For voltage resolution in the narrow intermediate range of 0.5 mV to 1 mV, where we observe somewhat broader features, the $\gamma$ may be considered to depend only on the number of points in the spectrum (it is possible to subsume this regime within the prior one). For coarser resolutions, and equivalently even broader features, a constant value of $\gamma$ is found to suffice. The quantitative dependencies are captured by the following formulas that are empirically found to apply to our synthetic dataset, and illustrated by the contours and curve in Fig. 6a,b.

For resolution = 0.0005, $$\gamma = 0.0816*\delta *\sqrt{n}$$

For resolution = 0.001, $$\gamma = 0.0086*\sqrt{n}$$

For resolution = 0.002, $$\gamma = 0.8$$

Here $\delta$ is the relative error after high pass filtering of the data, and n is the total number of data points.

It can be seen from Fig. 6 that the optimal $\gamma$ for the data from the experimental use cases of “Results and discussions: molecular junction” and “Results and discussions: MIS device” sections are also consistent with the above formulas. Further improvement in optimizing $\gamma$ could perhaps be achieved by using more experimental IETS data, and by incorporating better noise models into the synthetic data.

## Conclusion

We have applied Tikhonov Regularization based noise-filtering to compute the low-noise second derivative of representative DC I–V experimental measurements, as a tool for fast and simple numerical IET spectroscopy. This approach has been validated by demonstrating the match of IET spectra obtained using the algorithm to those from the standard second-harmonic lock-in measurement in two different systems, namely a molecular junction and an MIS diode. For both of these, we find that the algorithm replicates most, but not all, of the IET spectral peaks detected by lock-in measurement. It also finds a few peaks that the latter does not—but which do coincide with reported peaks in these systems. With an abundance of training data that would accompany real-life application, the algorithm should easily identify the material system in question.For that situation, where a standard IETS measurement will not be available as a reference, we have presented a method for automatic tuning of the algorithm parameters for optimal estimation of the derivative. Our method can greatly simplify IETS measurement hardware for analysis or sensor applications. It could also be useful for precision extraction of first derivatives, such as conductance, in a large range of applications. As usual, there do remain a few open questions. These concern the capability of the algorithm: to extract IETS lineshape modification due to metal clustering during device fabrication^34,35^; to extract IET spectra for the extremely large noise levels in the initial DC I–V^36^; and so forth.

## Supplementary Information

Supplementary Information.

## Data Availability

The datasets analysed during the current study available from the corresponding author on reasonable request.
