# Supplementary material for: Simplified inelastic electron tunneling spectroscopy based on low-noise derivatives
Source: Sci Rep. 2022 Nov 10;12:19216. doi: 10.1038/s41598-022-21302-4 (PMC9649763; doi:10.1038/s41598-022-21302-4)
Supplement: Supplementary file 1 — Supplementary Information. [file 41598_2022_21302_MOESM1_ESM.pdf]

Simplified Inelastic Electron Tunneling Spectroscopy Based on Low-Noise Derivatives - Supplementary Material

I. NOISE FILTERING ALGORITHM

We discuss in detail the numerical method based on the Tikhonov Regularization that can filter out noise while calculating the first and second derivatives from the data.

Let vector y be the measured experimental data for the current and x be its derivative, the conductivity. They are related by the matrix equation $\boldsymbol{A}x = y$, where $\boldsymbol{A}$ is the assembled matrix by finite difference scheme with a quadrature rule of integration. The method is based on minimizing the norm of second derivative vector Δx for smoothness, and at the same time keeping the computed x close to actual derivative from the measured data y. In other words, the norm of $\boldsymbol{A}x - y$ is kept close to δ, where δ is the noise level in the data and norm of Δx is also controlled such that solution is not too 'jittery'.

Mathematically, this can be expressed as the following. Solution x is the one that extremizes the functional J having two terms called as the Penalty term $R(x)$ and the Fidelity term F. The derivative of the measured data is computed in such fashion that it is the minimizer of the functional J.

$$J_\eta(x) = \phi(\boldsymbol{A}x - y) + \eta\psi(x), \tag{1}$$

which can be written in matrix form as,

$$J_\eta(x) = (\boldsymbol{A}x - y)^T(\boldsymbol{A}x - y) + \eta\Delta x^T\Delta x, \tag{2}$$

where, η is the tuning parameter, widely known as a regularization parameter that essentially is the outcome of well-known Tikhonov regularization [1].

The second term on the RHS of above equation, the 'fidelity term', indicates how close the computed value of x is close to the actual data, while the first term is the penalty is to be paid by going away from the actual solution. The balance between the two is to be maintained that can be controlled by η. Extremising the above action gives the equation of motion,

$$(\eta\Delta^T\Delta + \boldsymbol{A}^T A)x = \boldsymbol{A}^T y \tag{3}$$

Now, the rest of the method is about choosing the regularization parameter η. We use a rule for determining the regularization parameter η based on the balancing principle as described in [2]. Its relation to the L-curve criterion was discussed in [3]. In [4], the zero-crossing method was proposed, which finds $\eta > 0$ such that

$$\psi(x_n^\delta) = \gamma\eta\phi(x_n^\delta) \tag{4}$$

For a trial η, x is obtained from above equation from which the balancing condition of the penalty term with the fidelity term gives the next iterative value of η,

$$\eta = \frac{1}{\gamma}\frac{||\boldsymbol{A}x - y||_2}{||\Delta x^T\Delta x||_2} \tag{5}$$

where, γ is a user fixed positive constant. Initially a trial η is chosen, and above equation (5) and (3) is solved iteratively for η and x. A flowchart for the algorithm is shown in Fig. **S1**. A similar algorithm for a geophysical data-set has been applied by Roy et al [5].

II. MIS DEVICE - FABRICATION AND MEASUREMENT

An RCA cleaned n type Si substrate(100) of doping on the order of 10^{19}/cm^3 is used for the fabrication of the metal-insulator-semiconductor (MIS) tunneling device. The degenerate doping ensures conduction at cryogenic temperatures. The dielectric deposition is preceded by dipping the substrate in 2 % HF solution to remove the native oxide. Thereafter,2nm layer of HfO_2 is deposited with 21 cycles in an Atomic Layer Deposition (ALD) chamber at 200°C. Post deposition annealing is performed at 600°C for 5 min in N_2 environment. An annular window with a width of 100μm is opened using optical lithography to etch away the HfO_2 layer in BHF solution. Cr/Au of thickness 10/100 nm is then deposited by dc sputtering to make a ohmic contact with the highly doped Si substrate. This is in lieu of a 'bottom contact'. Finally for the circular 'top contact' of diameter 150μm atop the HfO_2, another level of optical lithography is performed followed by the metallization of Cr/Au of thickness 10/100nm, again by dc sputtering. The top view and cross-section of the structure is shown in Fig. **S2**.

Post wire bonding, IETS measurements were performed inside a cryostat at a temperature of 10K. This utilizes a home-made experimental setup, which uses an op-amp based adder for the dc + small-signal ac input to the device-under-test (DUT) as shown in Fig. 1 in the main manuscript; and, a lock-in amplifier to detect the second harmonic signal, which is proportional to d^2I/dV^2. The small-signal ac signal has amplitude 10 mV and frequency 42 Hz. This is added to a DC signal with a ramp rate of 0.2 mV/s and a step size of 1 mV. Noise reduction is effected by utilizing the input analog low pass filter of the lock-in amplifier - with a time constant of 1 second, as well as the synchronous filter option. Further, we have applied some smoothing within our plotting software for clearer delineation of the IETS peaks. We note here that the lock-in amplifier outputs the signal amplitude independent

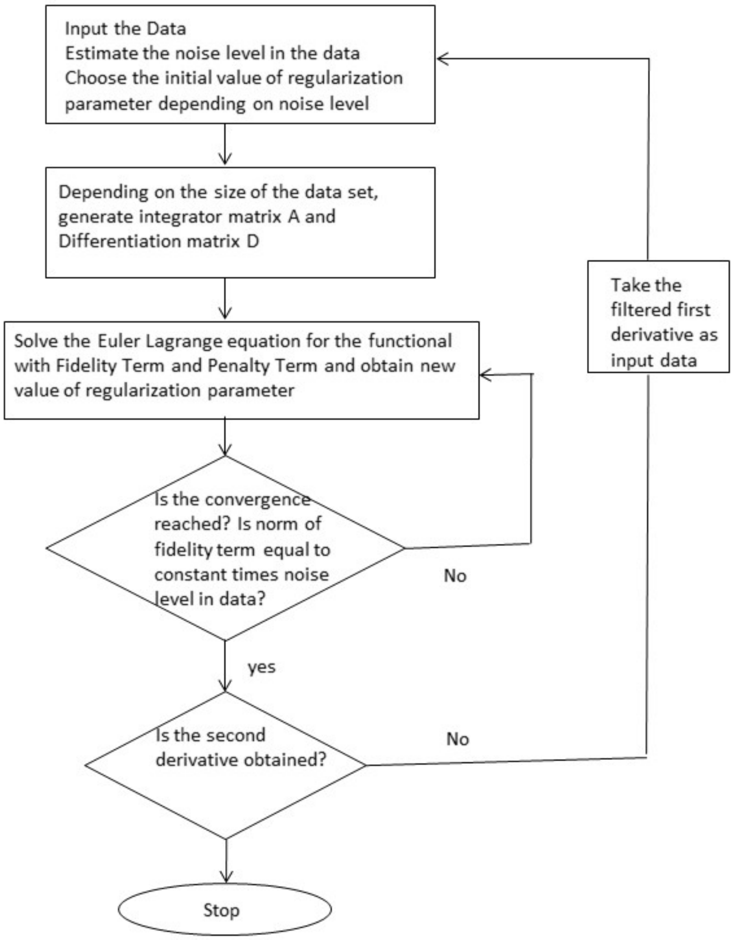

Fig. **S1**: Flowchart for the low-noise derivative (noise-filtering) algorithm

of its phase; it is therefore, always positive. Fig. **S3** shows a comparison of the conductance of this MIS tunneling device obtained by two methods. One, by application of the noise-filtering algorithm to the DC I-V data; and two, by small-signal measurement with lock-in detection of the first harmonic, which is proportional to the first derivative (i.e. conductance).

III. DERIVATIVE AUTOMATION

The synthetic data used here was generated as follows. It is observed (see Fig. 4 in the main manuscript) that IET spectra can be expressed as a sum of gaussians. Therefore, the synthetic data is constructed similarly. A sum of gaussians was generated using the random() functions in python. This is the 'reference' IETS data for this exercise. The number of gaussians was taken to be a random integer between 10 and 30. The centers of the gaussians were chosen to be in the same range as the defined X-axis. The amplitudes were chosen to be random values in the range [1e-9, 2e-9]. The σ of the gaussians were also selected from a fixed range, depending on the expected feature size (which can be thought to change as per the resolution in measurements).

After the sum of gaussians was obtained, it was integrated twice (using the rectangular rule) to get the synthetic data. To this synthetic 'noise-free I-V data, we added noise with various amplitudes for different numerical experiments. In keeping with our expectation of real systems, we added Additive White Gaussian Noise (AWGN) as well as colored noise - both pink ($1/f$ noise) and red ($1/f^2$).

The methodology is illustrated in Fig. **S4**. IETS obtained by application of the algorithm to the synthetic noisy I-V data is compared, in Fig. **S5**, to the reference viz. the starting (noise-free) sum of gaussians.

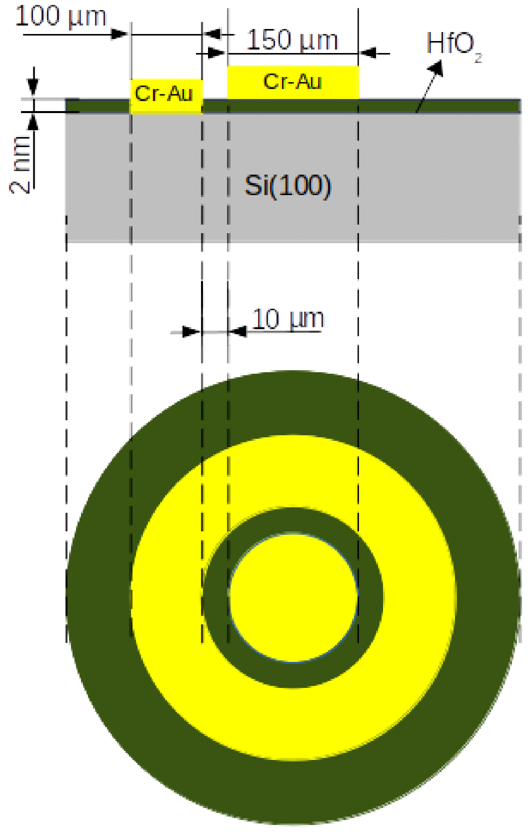

Fig. **S2**: Schematic diagram of MIS tunneling device - top and cross-sectional views

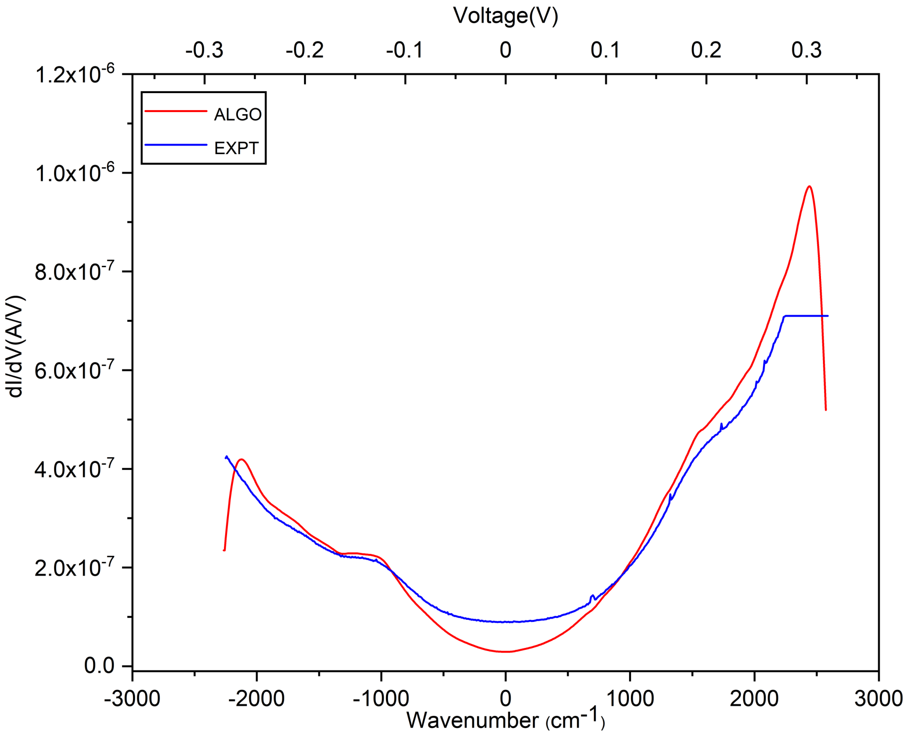

Fig. **S3**: dI/dV vs. V obtained from: small-signal lock-in measurement; and, application of the noise-filtering algorithm to measured dc I-V

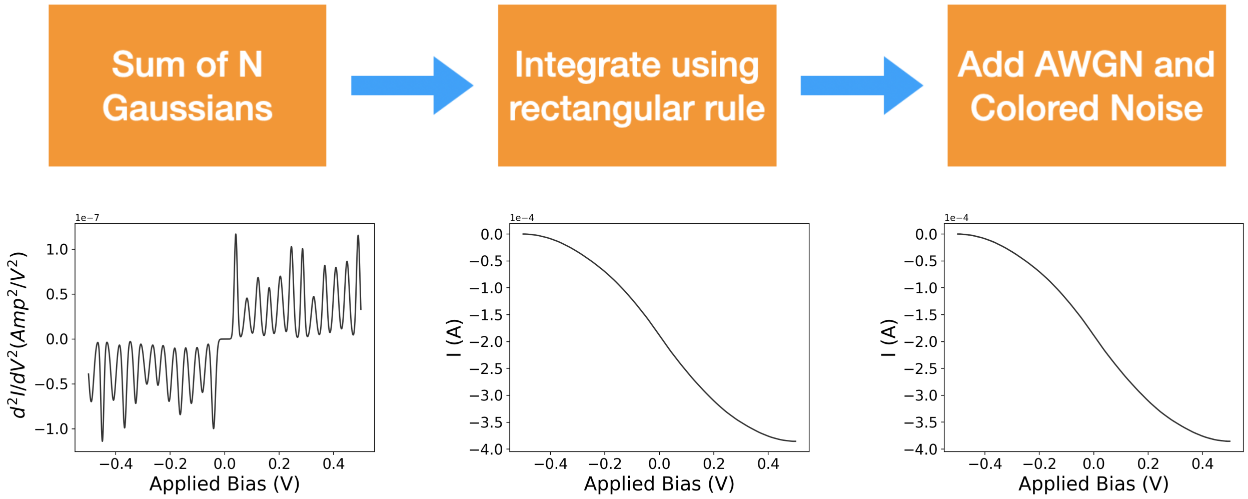

Fig. **S4**: (Top) Schematic flowchart for generation of synthetic noisy I-V data. (Bottom) Illustration of successive steps, namely: (left) generation of synthetic noise-free IETS data as sum of Gaussian peaks; (middle) integration of said IETS data twice to obtain synthetic noise-free I-V; (right) addition of white and colored noise to obtain synthetic noisy I-V.

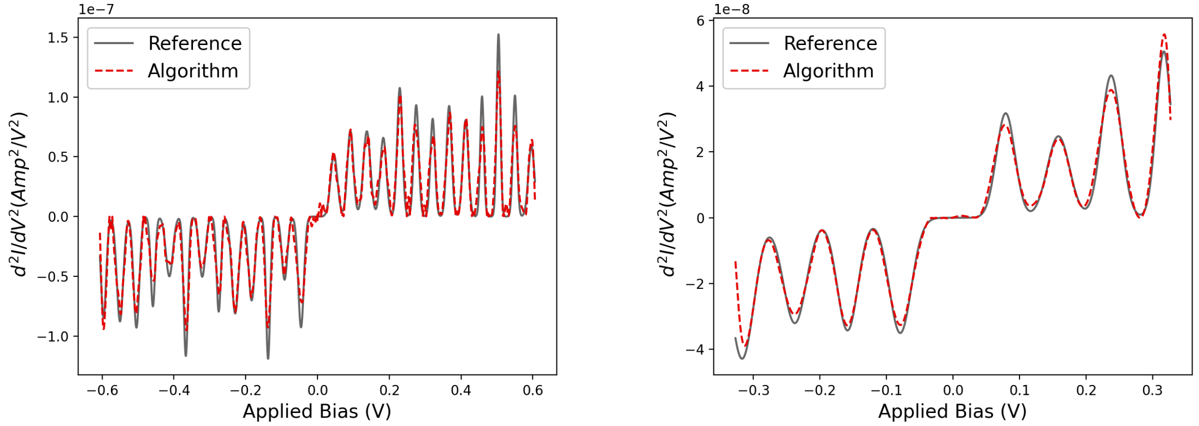

Fig. **S5**: Comparison of the reference data, viz. the noise-free synthetic IETS (d^2I/dV^2 vs. V), to the IETS obtained from application of the noise-filtering algorithm, for: (left) resolution = 0.005; and, (right) resolution = 0.01

References

[1] A. N. Tikhonov, A. Goncharsky, V. Stepanov, and A. G. Yagola, Numerical methods for the solution of ill-posed problems. Springer Science and Business Media, 2013, vol. 328.

[2] K. Ito, B. Jin, and T. Takeuchi, “A regularization parameter for nonsmooth tikhonov regularization,” SIAM Journal on Scientific Computing, vol. 33, no. 3, pp. 1415–1438, 2011.

[3] T. Regińska, “A regularization parameter in discrete ill-posed problems,” SIAM Journal on Scientific Computing, vol. 17, no. 3, pp. 740–749, 1996.

[4] P. R. Johnston and R. M. Gulrajani, “A new method for regularization parameter determination in the inverse problem of electrocardiography,” IEEE Transactions on Biomedical Engineering, vol. 44, no. 1, pp. 19–39, 1997.

[5] I. G. Roy, “On computing first and second order derivative spectra,” Journal of Computational Physics, vol. 295, pp. 307–321, 2015.
